# SARS-CoV-2 Pandemic and Food Safety Oversight: Implications in Canada and Coping Strategies

**DOI:** 10.3390/foods10102241

**Published:** 2021-09-22

**Authors:** Sylvain Charlebois, Janet Music

**Affiliations:** Faculty of Agriculture, Dalhousie University, Halifax, NS B2X 3T5, Canada; sylvain.charlebois@dal.ca

**Keywords:** SARS-CoV-2, COVID-19, Canada, food safety, food safety oversight

## Abstract

The SARS-CoV-2 pandemic has created enormous societal disruptions in the Western world, including Canada, with serious implications for food safety. Since the start of the pandemic, many scholars have investigated the issue of food safety through different lenses. In this review, two research thrusts were identified, the epidemiology of the virus and food safety oversight. Both were challenged by the pandemic in Canada and elsewhere. In this paper, we first present how Canada experienced the pandemic. We then present how epidemiology and food safety oversight were affected by the virus and how the spread exposed gaps in Canada’s food safety system. We explain how Canada was not adequately prepared to face the food safety challenges posed by SARS-CoV-2. The review ends with an explanation on how risk perceptions will be altered by the pandemic in Canada and how food safety systems will adjust to better anticipate systemic risks in the future.

## 1. Introduction

### 1.1. SARS-CoV-2

The SARS-CoV-2 pandemic likely brought forth a new era of food safety in which risks are managed and assessed very differently. The SARS-CoV-2 crisis showed that food safety measures must be adjusted even if the science related to the virus itself is ambiguous. Based on the science we had at the time of the outbreak, there was no evidence to suggest that consuming food was a risk and could have been associated with SARS-CoV-2 [1,2,3]. 

Strong evidence suggests that SARS-CoV-2 originated in bats, while others dispute this [4]. How it transferred to humans, however, remains unknown [5,6,7]. Regardless, such an observation has pushed many scientists to suggest that animal science and zoonotic diseases are forever closely linked to human health. In fact, Ma et al. (2021) noted that urbanization has promoted the occurrence of zoonotic disease transmission and we should expect to see more pandemics in the future [8]. According to the Global Health Estimates produced by the WHO, 20% of all global deaths could be attributed to infectious transmissible diseases [9]. Of these, 50% of the top five diseases were zoonotic in origin, including SARS-CoV-2 [10,11].

While there is no indication that SARS-CoV-2 is a foodborne virus, the impact the pandemic had on everyday life was unprecedented [12,13]. It has had serious consequences on what and how people eat and has brought to light important questions on how we keep our food safe, as well as the people responsible for our food: everyone from farmers to grocery store employees [14]. SARS-CoV-2 also exposed a major tear in the Western world’s safety net even though food safety practices are more robust and rigorous than ever, especially in Canada [15].

Consumers’ relationship with food also changed because of the pandemic. According to Kuna (2020), purchasing and eating habits were altered around the world [16]. People were purchasing less often in person and consuming more home-cooked meals while managing with existing supplies at home. As for food safety, the same survey showed that most people were confident in the safety of the food supply and the ability of food producers to meet strict standards [17].

The pandemic also compelled many food companies to exchange with the market using new means, compromising the effectiveness of food safety practices they were following before the crisis. With the pandemic, it seems the world pivoted to try to reach a consumer confined to staying home [18]. As a result, food supply chains around the world, including Canada, were much more democratized, meaning that consumers now have access to many other links in the supply chain, from farm to retail [19]. Furthermore, while food companies “pivoted” to e-commerce to reach consumers and e-procurement to reach processors and farmers, “delivery intermediaries” “copivoted” with food firms to help them deliver and procure phone apps and/or internet websites. This was crucial to the ability of the many food enterprises to pivot, including farmers’ markets, processors and other food providers which would not normally interact daily with the consumer before the pandemic [20]. 

### 1.2. Supply Chains and Food Safety

The pandemic generated supply chain disruptions all over the globe. Nonetheless, the pandemic also has shown that global supply chains, coupled with appropriate international logistical policies, can be resilient [21]. These disruptions can make a food supply chain more vulnerable to breaches in food safety practices. What were seen as standard protocols prior to the pandemic became a challenge, with the overwhelming uncertainty of how public health officials were going to contain risks created by SARS-CoV-2. Some reports suggested that food fraud cases may have been on the rise around the world due to the pandemic, but no evidence was gathered in Canada [22].

How risks were perceived by the public changed in a significant way. The pandemic has also brought stress and despair to populations, and Canada is no exception. During the SARS-CoV-2 pandemic, national and local infections and deaths were tracked daily and communicated in real time via multimedia [23]. This likely affected how risks were perceived in everyday life, including embedded risks related to food, how food was consumed and where it was purchased. Citizens of most countries have endured extraordinary restrictions to try and contain viral spread, reduce the number of deaths and relieve the strain on frontline health-care workers. This pressure has changed how risks are communicated by authorities and among consumers through social media.

**Figure 1:**
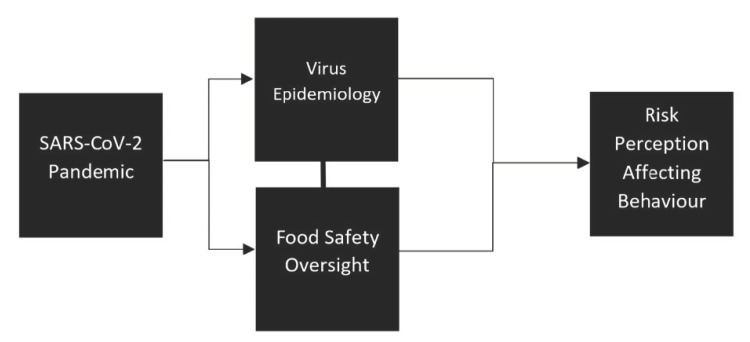
Effects of pandemic on consumers’ risk perception and behaviour.

Although the SARS-CoV-2 pandemic disrupted the Canadian food system, little is known about the degree to which consumers are connecting the virus to various aspects of the food safety system in the country, and how policy could be impacted moving forward [24] (see Figure 1). For this review, we first look at Canada’s food safety reality before and during the pandemic. We then look at the scholarly work conducted on food safety linked to the pandemic around the world, by considering three main research thrusts in food safety literature: food packaging, food safety training and surveillance and risk perception. Finally, we attempt to capture important findings which can influence food safety policies in a post-pandemic era.

## 2. SARS-CoV-2 in Canada

The pandemic affected Canada similarly to other parts of the Western World [25], but its food economy is unique in some facets. For one, Canada’s food distribution landscape is dominated by four companies, coupled with a few regional players. The main grocers are Loblaw, Sobeys, Walmart and Costco [26]. Being one of the largest countries in the world with a low population density in many regions, distribution costs are typically much higher in Canada than elsewhere around the world. Demand-side shocks came from consumers who stockpiled early in the pandemic, emptying shelves and buying many non-perishables such as dry pasta, canned goods and other center-of-the-store products [27,28]. The supply chain responded relatively well as most empty shelves were replenished within 5 to 7 days following the start of initial lockdowns [29].

As in many other Western countries, closures of restaurants in Canada and other food service businesses clearly caused a decline in expenditures on food consumed away from home, adding more pressure on retail. In Canada, the food service sector represented about 35% of all the food purchased in the country, pre-pandemic [30]. That percentage dropped to as low as 9% at the height of the panic-buying period [31].

Supply shocks were tempered by the fact that borders remained largely open for food products, which never really compromised Canada’s food security. Government interventions to secure access to food to their populations had adverse impacts in the form of negative international spillover effects in some parts of the world, but not in Canada [32]. Several countries imposed trade restrictions to avoid possible domestic supply shortages. In Canada, market access was never really compromised due to an open-border approach which was aligned with other major trading partners, like the United States. Canada is a major agri-food exporter, and the agriculture and food sector is an important component of the Canadian economy. Many commodities Canada produces are for export markets, and we import many processed goods. The United States is Canada’s most important trading partner in agri-food, which makes the border between the two countries even more critical [33].

Supply shocks due to restaurant closures generated more waste across the supply chain. Farmers who were growing crops for the food service sector were suddenly stuck with massive amounts of commodities. Products ranged from horticultural produce, to dairy, to mushrooms and more [34,35].

At the household level, things were similar. Demand shocks led to more food waste across the system [36]. Some of the household changes precipitated by the onset of the SARS-CoV-2 pandemic have enhanced the efficiency of the household food production process and reduced the overall amount of food wasted by consumers. In Canada, specifically, it was argued that both food literacy and gardening rates increased due to consumers staying home for longer periods [37,38].

Access to farm workers was also challenging due to travel restrictions. Canada typically hires about 65,000 foreign farm workers to support farming operations across the country [39]. Travel restrictions allowed Canadian farming operations to hire about 60% to 70% of what they would normally hire [34]. Some reports suggest farms had to forgo some harvest because of a labor shortage at farmgate. SARS-CoV-2-related protocols also slowed operations at the farm level across the country as farmers had to adapt and make changes to their operations on many levels. Nonetheless, agricultural trade in Canada grew by more than 10%, 12 months after the start of the pandemic. Most predictions had been suggesting the opposite [40].

The issue of the working poor also came up in Canada, as it did in many other countries. Many realized during the pandemic that frontline workers are frequently exposed to more risks and are underpaid. Calls for pay stipends, also known as “hero pay”, were frequent and came from regular citizens and policy makers [41]. The pandemic exposed inequities and inequalities throughout the food system, which impacted working conditions for employees with both normal and new food safety responsibilities [42]. It was argued that largely invisible workers in the food industry became suddenly quite visible and collectively underappreciated.

Throughout the pandemic, many food providers continued to retain access to consumers by delivering food or asking them to pick up food at certain locations. While many opted not to do anything, food providers in retail service and beyond continued to service the market by pivoting or copivoting. As a result, food offered to consumers was more diverse and wide-ranging, whether the service was offered virtually or in person [43].

However, Canada experienced many closures of meat processing plants, putting pressure on livestock farmers to find other ways to harvest their animals. Workers contracted the virus, resulting in the closure of over 15 plants for at least 14 days [44]. Plant closures due to food safety are not unusual in Canada [45]. The XL Foods recall in 2012, the largest recall in Canada’s history, forced the plant in Brooks, Alberta, to close for 27 days [46]. What is unique about SARS-CoV-2 is that many plants closed all at once, and in most cases, for a two-week period. Closures in sequence lasted over several months due to employees becoming sick. We estimate that 10 employees working in meat packing plants reportedly died from SARS-CoV-2-related complications during the pandemic [47]. The beef packing plant operated by Cargill in High River, Alberta, was at the center of the largest outbreak Canada experienced during the pandemic, resulting in over 1500 cases [48]. The Red Deer, Alberta, pork packing plant also became a source of concern a few months later when three employees died of health complications related to SARS-CoV-2.

Interestingly, the Canadian Food Inspection Agency (CFIA), responsible for food safety surveillance in Canada, was largely absent during the pandemic. Information conveyed to the Canadian public about food contamination related to SARS-CoV-2 was mostly coming from outside Canadian borders. The CDC provided most of the information to the Canadian public, leaving a risk communication gap for Canadians related to food products. The Public Health Agency of Canada did play an important role in risk communication, but its work looking at food was marginal.

Canada’s experience with the virus was like that of many other industrialized nations. However, most of the epidemiology and the knowledge developed related to the virus was not domestic or provided by Canadian-based institutions, which made risk assessment more challenging overall [49]. The scientific community is known to be effective in information sharing around the world, but knowledge gaps on how to contain the virus led to some confusion at times and have arguably affected food safety measures.

### 2.1. Virus Epidemiology

Eating habits for most changed because of the pandemic [50]. The market became more domesticated almost overnight as many were forced to stay home and cook meals, which was contrary to pre-pandemic habits, when many spent most of their time outside the home consuming food at restaurants and different outlets. That all changed in March 2020.

It was known even before the pandemic that this virus can spread through respiratory droplets from human-to-human contact. Food surfaces were also considered a way for the virus to spread and could also be carriers of the virus [51,52]. The food industry, particularly in processing, distribution, retail and service, dealt with concerns about surfaces and packaging, which influenced how facilities, stores and employees were managed. When the pandemic started, most food companies invested heavily in personal protection equipment and sanitation products to keep surfaces clean [53]. As research progressed, science was able to provide more clarity on how packaging and surfaces can act as vectors to spread the virus.

Both the World Health Organization (WHO) and Centers for Disease Control and Prevention (CDC) have acknowledged that there is no strong evidence of direct contamination of SARS-CoV-2 via food packaging surfaces or water ways [54], but risks cannot be ignored [55]. Yet, without any clear science, initial concerns over possible contamination of packaging materials overwhelmed decisions made by public health officials. Consumers were instructed to clean shopping bags and food packaging as they brough food into their homes. Retailers were continuously cleaning carts, doors, windows and other important sections of the grocery store to protect both the public and staff. Social distancing became the norm in public spaces, including grocery stores, on farms and in processing facilities [56].

Changes within the supply chain incurred by pivots and copivots created a situation in which plastics were overused. Some have claimed that there was a sharp increase in plastic waste from personal protective equipment such as masks and gloves, and from the massive return to using plastic shopping bags to prevent cross-contamination in food purchasing [57,58]. As explained earlier, lockdowns fostered e-commerce, curbside pick-up and deliveries, resulting in massive consumption of materials for containment and packaging. Few showed an interest in balancing risk mitigation regulations related to the virus with environmental protection policies [59].

The epidemiology of the virus was not clear, which impacted many other facets of our lives, including how consumers paid for food. Fueled by the fear of touching surfaces, the use of digital payments saw an increase during the pandemic [60]. In Western countries, the use of cash was no longer encouraged and digital payment or credit card payment was the preferred choice to pay, to limit contact with delivery partners [61,62]. Before the pandemic, a cashless economy was very much in progress, but SARS-CoV-2 has likely accelerated the process.

One noteworthy issue which came up during the pandemic was the role of the CFIA in risk communication. The CDC in the United States and the scientific community abroad have continuously sought to reassure the public that consuming food carries no risk of transmission of COVID-19, and that the virus itself would not survive on food packaging for an extended time. Canadian consumers were significantly concerned about contracting the virus directly from foods and food packaging [63]. Consumers have stigmatized portions of the food system because of not having sufficient or reliable epidemiological knowledge of the virus [64].

### 2.2. Food Safety Oversight

The pandemic also had a significant impact on oversight and surveillance of food safety risks [65,66]. While food itself is unlikely to be a source of coronavirus infection, the pandemic altered how public agencies inspect food and facilities, temporarily halting inspections of many facilities. Agencies had to conduct inspections using different means. Inspectors had to learn to use new technologies to conduct reviews offsite [67,68]. It is still unknown how effective these inspections were.

Djekic et al. (2021) noted that less than half of the food companies included in their global survey had documented any emergency plans associated with pandemics and health issues in place [69]. Pandemics were rarely considered threats before SARS-CoV-2.

Working conditions in some facilities became an issue early on during the pandemic. At the very beginning, many realized several facilities were ill-equipped to manage risks during a pandemic when workers themselves are at risk. Farms, meat-processing factories and distribution centers are designed to guarantee efficient production at minimal cost and irrespective of threats workers are exposed to. Employees undertake repetitive tasks in noisy conditions in proximity while participating in a quick-paced assembly line. Since many of these facilities are at low temperatures and SARS-CoV-2 spreads more easily in cold temperatures, risks are greater due to working conditions across the supply chain, especially in meat processing [70]. In addition, the noisy conditions necessitate shouting among workers, which is an action associated with a more intense transmission of the virus. All these aspects of work were not necessarily in the scope of inspections and training programs before the virus.

What also became obvious during the pandemic, which many scholars pointed out, is that many facilities in Canada are occupied predominantly by migrant and immigrant workers [71]. Not only do they often have to continue working due to their unfavorable socioeconomic status, but these workers will often also have low literacy and low levels of secondary school education, and little training [72]. Training and surveillance become challenging tasks when the workforce cannot appreciate or even understand the risks posed by the spread of a virus. With the many closures Canada experienced during the pandemic, it can be argued that many food safety regulators in provinces and across the country in Canada were not prepared for a pandemic. The human aspect of food safety was not as much of a focus for food safety regulators as it should have been, and SARS-CoV-2 made this painfully obvious in many countries, including Canada [73].

Outside of conditions in the plants, the pandemic also shed light on the living conditions of migrant workers across the supply chain. It was reported during the pandemic that many workers live in cramped unsanitary accommodation. In Canada, similarly, adverse living and working conditions have ensured the occupational transmission of SARS-CoV-2 among its agricultural workers [74]. For probably the first time, the scope of how enterprises managed risks had to include living conditions for workers, and not just operations and facilities which were frequently inspected.

During the pandemic, many food companies, farmers, processors and restaurants alike made responsive pivots to new market channels and buyers by leveraging relationships in local food supply chains. Short-circuit chains allow enterprises to develop direct, personal relationships with their supply chain partners and buyers [75]. When markets are dislocated, new relationships emerge and enable local organizations and food managers to leverage community networks to find necessary inputs, including labor, securing new buyers and expanding connections beyond the scope of the market they had prior to the pandemic. However, this also requires a different approach in food safety practices as targeted markets will have different expectations. Product design and assortment must be altered to better fit the needs of the new market enterprises pivoted to. As relationships change within the supply chain, so will food safety risks and measures taken to mitigate these risks [76].

More specifically, the food service industry was severely impacted by the crisis. Many establishments had to serve customers outside dining rooms, which added more complexity to food safety practices [77,78]. Many restaurants and different food service outlets were forced to serve consumers by delivering food, or by offering curbside pick-up services during lockdowns [79]. Catering was also severely affected by lockdowns [80]. This meant those restaurants required different food safety measures to maintain temperatures at an acceptable level. As a result, some research was conducted on food safety training in the food service industry [81,82].

Understanding what signals to look for to develop proper food safety programs was also a focus of research during the pandemic. In their comprehensive study of over 800 food companies, Djekic et al. (2021) recognized that staff awareness and hygiene are the two most important attributes in combatting SARS-CoV-2 [69]. Factors that were least salient were temperature checking of workers in food establishments and health protocols suggested by the WHO. Both were recognized as attributes with limited salience and significance [83]. In other words, actions by employees related to personal safety and hygiene are more significantly influential than enterprise-controlled protocols.

At the systems level, some research was conducted on federally coordinated systems which would allow for proactive risk surveillance. A nation such as Canada would require public health surveillance systems capable of monitoring the occurrence of serious illnesses as well as assessing the use, safety and effectiveness of treatments [68]. The Sentinel model in the United States is often mentioned as a significant case study. Such a system would routinely collect electronic health information, which would be coupled with a commitment to a network of diverse partners [84]. Such connections could fulfill the long-held promise of supporting essential public health needs during a crisis or a pandemic. This would potentially represent the next era of risk oversight in food safety.

## 3. Risk Perception Affecting Behavior

### Science Clarity

Many studies on the crisis have already provided evidence that public health events could enhance consumers’ food safety awareness and behavior, while consumers themselves focusing on pertinent information play an important role in improving knowledge and impacting behavior.

Around the world, there have been hotspots of infection and even more rigorous containment methods, such as lockdowns which limited the movement of all residents. These measures have had an impact on how people perceive risks. They have been implemented in high-risk locations. Throughout the pandemic, social distancing regulations have changed the way people interact with each other and there has been a significant increase in public health calls for hand hygiene, sanitation and self-isolation [85]. All these practices have altered social interactions and have changed the way business is conducted altogether. Facing the unknown, most jurisdictions wanted to prevent their health care system from becoming overwhelmed. However, such an approach gave way to economic uncertainty. The food industry was quickly protected from lockdowns and was allowed to carry on with their daily activities [86].

Science-based policies and regulations were skewed by our lack of understanding risks. Multiple studies were conducted on a variety of issues around the world to better understand how the virus impacted the health of several different groups [87,88]. Social media allowed populations to share information without any validation, which arguably led to some confusion on a variety of issues, including on the effectiveness of several public health measures.

One other aspect of risk perception and behavioral changes expected from the pandemic is how consumers will link animal diseases to food products they purchase every day. According to Attwood and Hajat (2020), the SARS-CoV-2 pandemic has already created a shift in public awareness of illnesses linked to animals and has resulted in short-term changes in patterns of meat consumption [47]. No studies have provided any evidence that a similar phenomenon is occurring in Canada. Past zoonotic outbreaks, such as SARS and swine flu, are also referred to, and we find that these led to comparable short-term reductions in meat consumption, a change in the type of meat chosen and potentially long-term impacts on consumer perceptions of the health risks associated with meat. For the livestock and meat industry, this cannot be overlooked.

Many of our food choices are driven by how we perceive risks. McCabe and Erdem postulated that death reminders, triggered by a pandemic in the case of SARS-CoV-2, can lead to different food choices [89]. How food is consumed, served, cooked, prepared and shared (or not) was largely influenced by the pandemic and how it impacted the way risks were perceived.

How risks are perceived will also be affected by who you are. Thilmany et al. (2021) postulated that consumer food concerns during the pandemic have unduly affected vulnerable people. Black respondents, as an example, were more concerned about contracting SARS-CoV-2 through food, with meat, grains and fruit seen as riskier [75]. These are important findings, suggesting that the stigma related to SARS-CoV-2 might further deepen standing racial inequalities related to nutrition and health [90]. However, it was largely inferred that the pandemic has generally pushed consumers to worry more about food safety than before [91,92].

Behaviors are always influenced by perceptions. In turn, how risks are perceived by a population is heavily affected by risk communication. Both industry and public agencies will communicate risks, and messaging becomes even more critical in times of crisis [93].

One key and obvious change brought on by the SARS-CoV-2 was an increase in handwashing practices at home and in public places. Public health officials repeatedly reminded the public to wash their hands. However, Thomas and Feng (2021) mentioned that consumers may not be connecting this practice to food safety, specifically. The practice of handwashing may be associated with a pandemic, more so than with food safety [78]. This could cause many to drop the habit after the pandemic.

During the pandemic, many argued that short-circuit food distribution, or localized food procurement strategies, would lessen risks of contamination. Some serious attention was given to diverting food systems and reviving locally made food and raw materials.

## 4. Discussion

### 4.1. Change in Scope

One of the ultimate challenges in crisis planning is developing food systems that are sufficiently resilient to continue functioning. For the most part, in the Western world, including Canada, food systems showed their resilience throughout the pandemic.

It can be argued that such a claim would force industry and regulators alike to broaden their scope of analysis when looking at food safety risks [26]. SARS-CoV-2 has reinforced the already pressing need for closer collaboration between animal and human health researchers, conservation practitioners, public health and environmental authorities. Without these collaborations, food safety measures cannot be as efficient, or at least as comprehensive.

From a global perspective, the immediate impact of the SARS-CoV-2 pandemic on the food supply chain was largely logistical. At first, food safety risks were implicit given how little we knew about the virus. Measures created logistical disruptions which have taken months to resolve.

It would be reasonable to expect that Canada’s food industry would remain resilient as most of the food businesses and the industry gained practical food safety know-how. The lessons learned from these disruptions are likely to heighten the supply chain responsiveness and resiliency once the pandemic is over. Nevertheless, potential structural changes exist, such as those that occurred after the many global food safety crises we have seen in the past, the effects of which on the food industry in Canada are less clear. SARS-CoV-2 brought forward minimal food safety risks but did trigger multiple structural changes to our economy and how we will consume food in the future.

Due to the economic changes and consumers working from home more often, food-at-home consumption will likely remain higher than its pre-pandemic levels, even long after the pandemic. How risks are managed will also need to change in that respect.

Most critically, food safety policies will often discriminate against small businesses, resulting in increasing exposure to supply chain disruptions and rates of shutdowns. When new food safety measures are implemented, small and medium sized businesses are often left behind. Canada’s listeria crisis is a good example [94]. How smaller businesses are supported when new risks emerge will be critical for the sustainability of the agri-food sector.

### 4.2. Human Capital and Data Science

It is easily foreseeable that the future of the food industry will be permanently changed due to the SARS-CoV-2 pandemic. The food industry in most parts of the world was labor intensive, and most operations were not capitalized enough to allow for more automation and the use of robotics. How risks are managed within food facilities will change. Food systems will be restructured and will give a greater priority to controlled environments in farming, processing and distribution, reducing the dependency on labor across food supply chains.

Bioanalytical tools to be used by industry and several participants of the food supply chain will be necessary to better anticipate risks. Stakeholders, from farm to fork, will need access to such technologies [95].

One thing we have learned throughout this pandemic is the power of data science and analytics. Open and predictable markets due to the use of more data science are critical to ease the distribution of food along supply chains and to ensure food can move to where it is needed. Analytical platforms can combine data on temperature, humidity and duration in the food chain to forecast pathogen infection and act before a contamination occurs.

Moving to a post-lockdown practice, public health surveillance will not only need to use more data science but will also depend more and more on the development of appropriate bioanalytical tools. This tactic may not only relate to screening of populations but also monitoring of foods, surfaces and surrounding environments. Technologies can be developed to support industry in the effort to make food supply chains work efficiently.

## 5. Conclusions

We will likely face more pandemics in the future. Unless fundamental changes are made to anticipate zoonotic diseases, much of the global response to these outbreaks will continue to be reactionary. The focus will continually be to contain the spread and rebuild the economy.

With data science, pandemic control needs synchronization among several stakeholders, including the farming community, epidemiologists, animal science researchers, wet market traders, exporters, local businesses and consumers. Connections among these important participants in the food supply chain will be critical for better data sharing and holistic risk assessment. Evidence-based policy advice requires data and, unfortunately, many stakeholders were flying blind for many parts of the pandemic and evidence has remained inconsistent during most of the pandemic, fostering doubt and uncertainty amongst consumers.

One can argue that SARS-CoV-2 has started a new era in which food safety oversight is more ubiquitous in the food industry. That era will likely be defined by how holistic risks are considered and managed by the food industry. It is no longer just about food safety but rather the integrity of the entire system. This covers several aspects, from risks in farm fields, to conditions in which workers live, to how the entire supply chain can cope with the closure of a massive economic sector, literally overnight.

At the very beginning of the crisis, the food industry was facing huge uncertainties regarding the presence of SARS-CoV-2 in food production and distribution. With more research and science, the industry’s path moving forward will likely be clearer. More importantly, industry can better anticipate how public health officials will manage risks related to a highly contagious virus. Pandemics are likely to happen again.

## Data Availability

This study did not report any new data.

## References

[1] Centers for Disease Control Coronavirus Disease 2019 (COVID-19). https://www.cdc.gov/coronavirus/2019-ncov/prevent-getting-sick/prevention.html/.

[2] Han S., Roy P.K., Hossain M.I., Byun K.-H., Choi C., Ha S.-D. (2021). COVID-19 pandemic crisis and food safety: Implications and inactivation strategies. Trends food Sci. Technol..

[3] Lacombe A., Quintela I., Liao Y.-T., Wu V.C.H. (2020). Food safety lessons learned from the COVID-19 pandemic. J. Food Saf..

[4] Rocha R., Aziz S.A., Brook C.E., Carvalho W.D., Cooper-Bohannon R., Frick W.F., Huang J.C.-C., Kingston T., López-Baucells A., Maas B. (2020). Bat conservation and zoonotic disease risk: A research agenda to prevent misguided persecution in the aftermath of COVID-19. Anim. Conserv..

[5] Mallapaty S. (2020). Meet the scientists investigating the origins of the COVID pandemic. Nature.

[6] Mallapaty S., Callaway E. (2021). What scientists do and don’t know about the Oxford–AstraZeneca COVID vaccine. Nature.

[7] Min S., Xiang C., Zhang X. (2020). Impacts of the COVID-19 pandemic on consumers’ food safety knowledge and behavior in China. J. Integr. Agric..

[8] Ma N.L., Peng W., Soon C.F., Noor Hassim M.F., Misbah S., Rahmat Z., Yong W.T.L., Sonne C. (2021). Covid-19 pandemic in the lens of food safety and security. Environ. Res..

[9] Dörks M., Jobski K., Hoffmann F., Douros A. (2021). Global COVID-19 pandemic and reporting behavior—An analysis of the Food and Drug Administration adverse events reporting system. Pharmacoepidemiol. Drug Saf..

[10] Aiyar A., Pingali P. (2020). Pandemics and food systems—Towards a proactive food safety approach to disease prevention & management. Food Secur..

[11] Ejeromedoghene O., Tesi J.N., Uyanga V.A., Adebayo A.O., Nwosisi M.C., Tesi G.O., Akinyeye R.O. (2020). Food security and safety concerns in animal production and public health issues in Africa: A perspective of COVID-19 pandemic era. Ethics Med. Public Health.

[12] Oliveira T.C., Abranches M.V., Lana R.M. (2020). (In)Segurança alimentar no contexto da pandemia por SARS-CoV-2. Cad. Saude Publica.

[13] D’Souza R., Ashraf R., Rowe H., Zipursky J., Clarfield L., Maxwell C., Arzola C., Lapinsky S., Paquette K., Murthy S. (2021). Pregnancy and COVID-19: Pharmacologic considerations. Ultrasound Obstet. Gynecol..

[14] Cable J., Jaykus L., Hoelzer K., Newton J., Torero M. (2020). The impact of COVID-19 on food systems, safety, and security—A symposium report. Ann. N. Y. Acad. Sci..

[15] Le Vallée J.-C., Charlebois S. (2015). Benchmarking Global Food Safety Performances: The Era of Risk Intelligence. J. Food Prot..

[16] Kuna A. (2020). Impact of Covid—19 on Food Purchasing, Eating Behaviors and Perceptions of Food Safety in Consumers of Telangana and Andhra Pradesh of India. Int. J. Agric. Environ. Biotechnol..

[17] Côté D., Durant S., MacEachen E., Majowicz S., Meyer S., Huynh A.-T., Laberge M., Dubé J. (2021). A rapid scoping review of COVID-19 and vulnerable workers: Intersecting occupational and public health issues. Am. J. Ind. Med..

[18] McCabe S., Erdem S. (2021). The influence of mortality reminders on cultural in-group versus out-group takeaway food safety perceptions during the COVID-19 pandemic. J. Appl. Soc. Psychol..

[19] Chenarides L., Manfredo M., Richards T.J. (2020). COVID-19 and Food Supply Chains. Appl. Econ. Perspect. Policy.

[20] Reardon T., Heiman A., Lu L., Nuthalapati C.S.R., Vos R., Zilberman D. (2021). “Pivoting” by food industry firms to cope with COVID-19 in developing regions: E-commerce and “copivoting” delivery intermediaries. Agric. Econ..

[21] Deconinck K., Avery E., Jackson L.A. (2020). Food Supply Chains and Covid-19: Impacts and Policy Lessons. EuroChoices.

[22] Golembeski C.A., Irfan A., Dong K.R. (2020). Food Insecurity and Collateral Consequences of Punishment Amidst the COVID-19 Pandemic. World Med. Health policy.

[23] Stockwell T., Andreasson S., Cherpitel C., Chikritzhs T., Dangardt F., Holder H., Naimi T., Sherk A. (2021). The burden of alcohol on health care during COVID-19. Drug Alcohol Rev..

[24] McFadden B.R., Malone T., Kecinski M., Messer K.D. (2021). COVID-19 Induced Stigma in U.S. Consumers: Evidence and Implications. Am. J. Agric. Econ..

[25] Hobbs J.E. (2020). Food supply chains during the COVID-19 pandemic. Can. J. Agric. Econ. Rev. Can. d’Agroecon..

[26] Zhang W., He H., Zhu L., Liu G., Wu L. (2021). Food Safety in Post-COVID-19 Pandemic: Challenges and Countermeasures. Biosensors.

[27] Du H., Yang J., King R.B., Yang L., Chi P. (2020). COVID-19 Increases Online Searches for Emotional and Health-Related Terms. Appl. Psychol. Health Well-Being.

[28] Taylor S. (2021). Understanding and managing pandemic-related panic buying. J. Anxiety Disord..

[29] Gupta A., Zhu H., Doan M.K., Michuda A., Majumder B. (2020). Economic Impacts of the COVID−19 Lockdown in a Remittance-Dependent Region. Am. J. Agric. Econ..

[30] Jha A. (2021). A strategic apparoach for manageing COVID-19 crisis: A food delivery industry perspective. Acad. Mark. Stud. J..

[31] Richards T.J., Rickard B. (2020). COVID-19 impact on fruit and vegetable markets. Can. J. Agric. Econ. Can. d’Agroecon..

[32] Glauber J., Laborde Debucquet D., Martin W., Vos R. (2020). COVID-19: Trade Restrictions are Worst Possible Response to Safeguard Food Security.

[33] Zuber S., Brüssow H. (2020). COVID 19: Challenges for virologists in the food industry. Microb. Biotechnol..

[34] Rude J. (2020). COVID-19 and the Canadian cattle/beef sector: Some preliminary analysis. Can. J. Agric. Econ. Can. d’Agroecon..

[35] Laborde D., Martin W., Vos R. (2021). Impacts of COVID-19 on global poverty, food security, and diets: Insights from global model scenario analysis. Agric. Econ..

[36] Roe B.E., Bender K., Qi D. (2020). The Impact of COVID-19 on Consumer Food Waste. Appl. Econ. Perspect. Policy.

[37] Mullins L., Charlebois S., Finch E., Music J. (2021). Home Food Gardening in Canada in Response to the COVID-19 Pandemic. Sustainability.

[38] Music J., Finch E., Gone P., Toze S., Charlebois S., Mullins L. (2021). Pandemic Victory Gardens: Potential for local land use policies. Land Use Policy.

[39] Brewin D.G. (2020). The impact of COVID-19 on the grains and oilseeds sector. Can. J. Agric. Econ. Can. d’Agroecon..

[40] Barichello R. (2021). Revisiting the effects of the COVID-19 pandemic on Canada’s agricultural trade: The surprising case of an agricultural export boom. Can. J. Agric. Econ. Can. d’Agroecon..

[41] Parks C.A., Nugent N.B., Fleischhacker S.E., Yaroch A.L. (2020). Food System Workers are the Unexpected but Under Protected COVID Heroes. J. Nutr..

[42] Rossiter K., Godderis R. (2020). Essentially invisible: Risk and personal support workers in the time of COVID-19. Sociol. Health Illn..

[43] Lurie N., Keusch G.T., Dzau V.J. (2021). Urgent lessons from COVID 19: Why the world needs a standing, coordinated system and sustainable financing for global research and development. Lancet.

[44] McEwan K., Marchand L., Shang M.Z. (2021). The Canadian pork industry and COVID-19: A year of resilience. Can. J. Agric. Econ. Can. d’Agroecon..

[45] Hailu G. (2021). COVID-19 and food processing in Canada. Can. J. Agric. Econ. Can. d’Agroecon..

[46] Charlebois S., Von Massow M., Pinto W. (2014). Food Recalls and Risk Perception: An Exploratory Case of the XL Foods and the Biggest Food Recall in Canadian History. J. Food Prod. Mark..

[47] Attwood S., Hajat C. (2020). How will the COVID-19 pandemic shape the future of meat consumption?. Public Health Nutr..

[48] Treble P., Cattermore L. (2020). The Aftermath of COVID.

[49] Mallapaty S. (2021). Where did COVID come from? Five mysteries that remain. Nature.

[50] Galali Y. (2021). The impact of COVID-19 confinement on the eating habits and lifestyle changes: A cross sectional study. Food Sci. Nutr..

[51] Mullis L., Saif L.J., Zhang Y., Zhang X., Azevedo M.S.P. (2012). Stability of bovine coronavirus on lettuce surfaces under household refrigeration conditions. Food Microbiol..

[52] Maragoni-Santos C., Serrano Pinheiro de Souza T., Matheus J.R.V., de Brito Nogueira T.B., Xavier-Santos D., Miyahira R.F., Costa Antunes A.E., Fai A.E.C. (2021). COVID-19 pandemic sheds light on the importance of food safety practices: Risks, global recommendations, and perspectives. Crit. Rev. Food Sci. Nutr..

[53] Pitts E.R., Witrick K. (2021). Brewery Packaging in a Post-COVID Economy within the United States. Beverages.

[54] Centers for Disease Control What Food and Grocery Pick-Up and Delivery Drivers Need to Know about COVID-19. https://www.cdc.gov/coronavirus/2019-ncov/community/organizations/food-grocery-drivers.html.

[55] Galanakis C.M. (2020). The Food Systems in the Era of the Coronavirus (COVID-19) Pandemic Crisis. Foods.

[56] Swinnen J., Vos R. (2021). COVID-19 and impacts on global food systems and household welfare: Introduction to a special issue. Agric. Econ..

[57] Klemeš J.J., Van Fan Y., Tan R.R., Jiang P. (2020). Minimising the present and future plastic waste, energy and environmental footprints related to COVID-19. Renew. Sustain. Energy Rev..

[58] Fadare O.O., Okoffo E.D. (2020). Covid-19 face masks: A potential source of microplastic fibers in the environment. Sci. Total Environ..

[59] Gorrasi G., Sorrentino A., Lichtfouse E. (2020). Back to plastic pollution in COVID times. Environ. Chem. Lett..

[60] Popkin B.M., Du S., Green W.D., Beck M.A., Algaith T., Herbst C.H., Alsukait R.F., Alluhidan M., Alazemi N., Shekar M. (2020). Individuals with obesity and COVID-19: A global perspective on the epidemiology and biological relationships. Obes. Rev..

[61] Nguyen T.H.D., Vu D.C. (2020). Food Delivery Service During Social Distancing: Proactively Preventing or Potentially Spreading Coronavirus Disease-2019?. Disaster Med. Public Health Prep..

[62] Mehrolia S., Alagarsamy S., Solaikutty V.M. (2020). Customers response to online food delivery services during COVID-19 outbreak using binary logistic regression. Int. J. Consum. Stud..

[63] Walker T.R., McGuinty E., Charlebois S., Music J. (2021). Single-use plastic packaging in the Canadian food industry: Consumer behavior and perceptions. Humanit. Soc. Sci. Commun..

[64] Kitz R., Walker T., Charlebois S., Music J. (2021). Food packaging during the COVID-19 pandemic: Consumer perceptions. Int. J. Consum. Stud..

[65] Ramsey A.F., Goodwin B.K., Hahn W.F., Holt M.T. (2021). Impacts of COVID-19 and Price Transmission in U.S. Meat Markets. Agric. Econ..

[66] Mukhtar A., Shukry M., Bannan D. (2021). Safe handling and delivery of biological medications during the COVID-19 pandemic. J. Clin. Pharm. Ther..

[67] Ranaei V., Pilevar Z., Hosseini H. (2020). Food Safety Practices in COVID-19 Pandemic. J. Food Qual. Hazards Control.

[68] Cocoros N.M., Fuller C.C., Adimadhyam S., Ball R., Brown J.S., Dal Pan G.J., Kluberg S.A., Lo Re 3rd V., Maro J.C., Nguyen M. (2021). A COVID-19-ready public health surveillance system: The Food and Drug Administration’s Sentinel System. Pharmacoepidemiol. Drug Saf..

[69] Djekic I., Nikolić A., Uzunović M., Marijke A., Liu A., Han J., Brnčić M., Knežević N., Papademas P., Lemoniati K. (2021). Covid-19 pandemic effects on food safety—Multi-country survey study. Food Control.

[70] Reid A., Ronda-Perez E., Schenker M.B. (2020). Migrant workers, essential work, and COVID-19. Am. J. Ind. Med..

[71] Rosenbaum L. (2021). Escaping Catch-22—Overcoming Covid Vaccine Hesitancy. N. Engl. J. Med..

[72] Ellison B., McFadden B., Rickard B.J., Wilson N.L.W. (2020). Examining Food Purchase Behavior and Food Values during the COVID-19 Pandemic. Appl. Econ. Perspect. Policy.

[73] Larocque C., Foth T. (2021). Which lives are worth saving? Biolegitimacy and harm reduction during COVID-19. Nurs. Inq..

[74] Arora S., Majumder M. (2021). Where is my home?: Gendered precarity and the experience of COVID-19 among women migrant workers from Delhi and National Capital Region, India. Gender Work. Organ..

[75] Thilmany D., Canales E., Low S.A., Boys K. (2020). Local Food Supply Chain Dynamics and Resilience during COVID-19. Appl. Econ. Perspect. Policy.

[76] Ruggeri G., Mazzocchi C., Corsi S. (2016). Urban Gardeners’ Motivations in a Metropolitan City: The Case of Milan. Sustainability.

[77] de Freitas R.S.G., Stedefeldt E. (2020). COVID-19 pandemic underlines the need to build resilience in commercial restaurants’ food safety. Food Res. Int..

[78] Thomas M.S., Feng Y. (2021). Food Handling Practices in the Era of COVID-19: A Mixed-Method Longitudinal Needs Assessment of Consumers in the United States. J. Food Prot..

[79] Omar S.S. (2020). Impact of pandemic crisis: COVID-19 on food safety knowledge, attitudes and practices among food workers in Jordan. Eurasian J. Biosci..

[80] Mayurnikova L., Koksharov A., Krapiva T. (2020). Food safety practices in catering during the coronavirus COVID-19 pandemic. Foods Raw Mater..

[81] Ceryes C., Robinson J., Biehl E., Wirtz A.L., Barnett D.J., Neff R. (2021). Frequency of Workplace Controls and Associations With Safety Perceptions Among a National Sample of US Food Retail Workers During the COVID-19 Pandemic. J. Occup. Environ. Med..

[82] Mohammadi-Nasrabadi F., Salmani Y., Esfarjani F. (2021). A quasi-experimental study on the effect of health and food safety training intervention on restaurant food handlers during the COVID-19 pandemic. Food Sci. Nutr..

[83] Çakır M., Li Q., Yang X. (2020). COVID-19 and fresh produce markets in the United States and China. Appl. Econ. Perspect. Policy.

[84] Epstein J., Smid W.M., Wendel S., Somuah D., Burnouf T. (2021). Use of COVID-19 convalescent plasma in low- and middle-income countries: A call for ethical principles and the assurance of quality and safety. Vox Sang..

[85] Kuhn E.J., Walker G.S., Wright J., Whiley H., Ross K.E. (2021). Public health challenges facing Environmental Health Officers during COVID-19: Methamphetamine contamination of properties. Aust. N. Z. J. Public Health.

[86] Larue B. (2021). COVID-19 and labor issues: An assessment. Can. J. Agric. Econ. Can. d’Agroecon..

[87] Palalioglu R.M., Mahammadaliyeva A., Erbiyik H.I., Muhcu M. (2021). COVID-19 in third trimester may not be as scary as you think, it can be innocent: Evaluating vertical transmission from a COVID-19 positive asymptomatic pregnant woman with early membrane rupture. J. Obstet. Gynaecol. Res..

[88] Vardoulakis S., Sheel M., Lal A., Gray D. (2020). COVID-19 environmental transmission and preventive public health measures. Aust. N. Z. J. Public Health.

[89] (2020). Food safety risk during the pandemic. Food Sci. Technol..

[90] Pfaar O., Klimek L., Jutel M., Akdis C.A., Bousquet J., Breiteneder H., Chinthrajah S., Diamant Z., Eiwegger T., Fokkens W.J. (2021). COVID-19 pandemic: Practical considerations on the organization of an allergy clinic—An EAACI/ARIA Position Paper. Allergy.

[91] Charlebois S., Vandertuin T. (2021). Food Safety Economics in the COVID-19 Pandemic. J. Food Res..

[92] Dumas B., Lee S.H., Harris D., Pomeroy M., Yaroch A., Blanck H. (2021). Characteristics Associated With Self-Reported Worry Among Adults About Food Availability and Food Safety During the COVID-19 Pandemic—United States, June 2020. Curr. Dev. Nutr..

[93] Charlebois S., Summan A. (2015). A risk communication model for food regulatory agencies in modern society. Trends Food Sci. Technol..

[94] Charlebois S., Horan H. (2010). Institutional and relational determinants in high- and medium-extent food product crises: The inner perspective of a public health crisis. Int. J. Environ. Health Res..

[95] Rizou M., Galanakis I.M., Aldawoud T.M.S., Galanakis C.M. (2020). Safety of foods, food supply chain and environment within the COVID-19 pandemic. Trends Food Sci. Technol..

